# DipM is required for peptidoglycan hydrolysis during chloroplast division

**DOI:** 10.1186/1471-2229-14-57

**Published:** 2014-03-06

**Authors:** Shin-ya Miyagishima, Yukihiro Kabeya, Chieko Sugita, Mamoru Sugita, Takayuki Fujiwara

**Affiliations:** 1 Center for Frontier Research, National Institute of Genetics, 1111 Yata, Mishima, Shizuoka 411-8540, Japan; 2 Center for Gene Research, Nagoya University, Chikusa-ku, Nagoya 464-8602, Japan

**Keywords:** Chloroplast division, DipM, Endosymbiosis, Glaucophytes, Peptidoglycan

## Abstract

**Background:**

Chloroplasts have evolved from a cyanobacterial endosymbiont and their continuity has been maintained over time by chloroplast division, a process which is performed by the constriction of a ring-like division complex at the division site. The division complex has retained certain components of the cyanobacterial division complex, which function inside the chloroplast. It also contains components developed by the host cell, which function outside of the chloroplast and are believed to generate constrictive force from the cytosolic side, at least in red algae and Viridiplantae. In contrast to the chloroplasts in these lineages, those in glaucophyte algae possess a peptidoglycan layer between the two envelope membranes, as do cyanobacteria.

**Results:**

In this study, we show that chloroplast division in the glaucophyte *C. paradoxa* does not involve any known chloroplast division proteins of the host eukaryotic origin, but rather, peptidoglycan spitting and probably the outer envelope division process rely on peptidoglycan hydrolyzing activity at the division site by the DipM protein, as in cyanobacterial cell division. In addition, we found that DipM is required for normal chloroplast division in the moss *Physcomitrella patens*.

**Conclusions:**

These results suggest that the regulation of peptidoglycan splitting was essential for chloroplast division in the early evolution of chloroplasts and this activity is likely still involved in chloroplast division in Viridiplantae.

## Background

Chloroplasts arose more than one billion years ago when a cyanobacterium became an endosymbiont in a eukaryotic cell. Now, several lines of evidence have come to suggest that a unique endosymbiotic event gave rise to the chloroplasts of Glaucophyta (glaucophyte algae), Rhodophyta (red algae) and Viridiplantae (green algae, charophyte algae and land plants). All other photosynthetic eukaryotes acquired chloroplasts by subsequent endosymbiosis in which a green or red alga was integrated into a previously non-photosynthetic eukaryote [1]. Over time, most of the genes once present in the endosymbiont have been lost or transferred to the host nuclear genome; those that are still used by the chloroplast are translated by the host and targeted back into the organelle, where they perform their functions. Consistent with this scenario, chloroplasts replicate by the division of the preexisting organelle and chloroplast division is performed by proteins encoded in the nuclear genome [2-6]. It is believed that this regulation of chloroplast division by the eukaryotic host cell ensured permanent inheritance of the chloroplasts during the course of cell division and from generation to generation [7].

Earlier electron microscopy studies established that chloroplast division (except in glaucophytes; see below) is performed by the simultaneous constriction of the inner and the outer envelope at the division site. In addition, structures surrounding the division site have been identified on both the cytosolic and stromal sides of the envelope membranes, i.e. the outer and the inner plastid-dividing (PD) rings [8]. Recent molecular genetic and biochemical studies have shown that chloroplast division is performed by the constriction of a large protein complex at the division site that encompasses both the inside and the outside of the two envelopes. The division complex involves proteins derived from the cytokinetic machinery of cyanobacteria (the functions on the stromal side of the division site) and proteins that originated from the eukaryotic host cell (the functions mainly on the cytosolic side). At the chloroplast division site, nuclear encoded, the cyanobacteria-descended FtsZ protein self-assembles into a ring structure, which in turn leads to the recruitment of other chloroplast division proteins of eukaryotic host origin. FtsZ ring formation is regulated by the cyanobacteria-descended ARC6, MinD and MinE proteins in a similar manner to cyanobacterial cell division. Chloroplast division proteins of the eukaryotic host origin have been integrated in a stepwise manner into the division complex during evolution. The dynamin-related protein DRP5B and the outer PD ring (composed of glucan filaments) were added before the split of red algae and Viridiplantae (i.e. they are conserved in both lineages). In contrast, the others, including the outer envelope spanning proteins PDV1 and PDV2 and the inner envelope-spanning protein MCD1 are unique to land plants [2-6].

This account of the mechanism of chloroplast division is mainly based on studies using red algae and land plants. In contrast to these lineages, the chloroplasts of the glaucophyte algae possess a peptidoglycan (PG) layer between the two envelope membranes, as do bacteria. Evolutionary studies suggest that the glaucophyte algae were the earliest to branch off from the common ancestor of Plantae, prior to the divergence of the red algae and Viridiplantae [1]. Chloroplast division in the glaucophyte alga *C. paradoxa* involves FtsZ ring formation on the stromal side of the division site, as in cyanobacteria and chloroplasts of the other lineages [9,10]. A structure similar to the inner PD ring has been detected by electron microscopy [11,12]. However, the outer PD ring was not evident [11,12] and the chloroplast division genes of eukaryotic host origin, including *drp5B*, were not found in the EST database [13]. Thus, the mechanism of the glaucophyte chloroplast division other than the portion working inside the chloroplast (i.e. the inner envelope membrane and the stromal side) appears to be different from that in other lineages.

**Figure 1:**
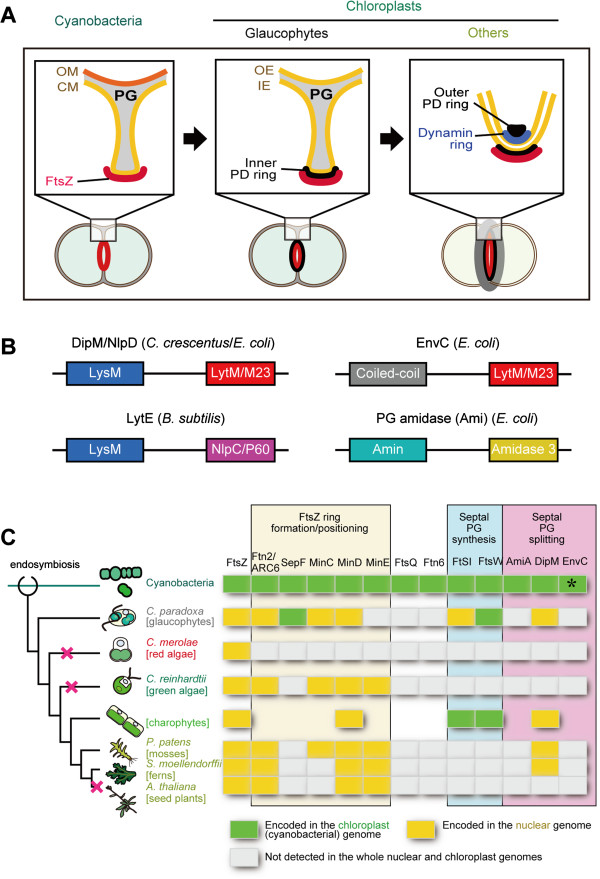
**Schematic view of the cyanobacterial and chloroplast division complexes and distribution of the chloroplast division proteins of cyanobacterial origin. (A)** Schematic comparison of the division complex among cyanobacteria, along with the chloroplasts of glaucophytes and those in other lineages. Glaucophyte chloroplasts have a PG between the inner- and the outer-envelope membrane, as do cyanobacteria. To allow the outer envelope membrane constrict, the PG layer at the division site has to be cut from the outermost site (a process called PG splitting), as in bacterial cell division. CM, cytoplasmic membrane; IE, inner envelope membrane; OE, outer envelope membrane; OM, outer membrane; PG, peptidoglycan layer. **(B)** Domain organization of DipM/NlpD, EnvC, LytE and PG amidases (Ami) which are involved in PG splitting in bacteria. DipM/NlpD and LytE usually contain three to five repeats of the LysM motif, but only one LysM motif is shown. The N-terminal LysM, coiled-coil or Amin domain targets the protein to the septum and the C-terminal LytM/M23, NlpC/P60 or Amidase 3 domain has a hydrolase activity. **(C)** Distribution of the chloroplast division proteins of cyanobacterial origin (updated from [32]). A certain lineages of photosynthetic eukaryotes possess DipM homologs probably descended from a cyanobacterial ancestor of chloroplasts. The branch lengths do not represent the phylogenetic distance. The red cross marks on the nodes indicate the deduced timing of loss of DipM. The GenBank accession numbers of the amino acid sequences are summarized in Additional file 1: Table S1. EnvC was found in some of the cyanobacterial species (e.g. GI:427708140 in *Nostoc* sp. PCC 7107), but not in *S. elongatus*. The data on the charophytes are not based on a single species but a combination of species.

In glaucophyte chloroplast division, the inner envelope membrane starts to constrict earlier than the outer envelope membrane does, and this is accompanied by an ingrowth of the PG layer at the division site, reminiscent of the cell division of bacteria. Therefore, the gap between the two envelopes at the division site becomes much larger than in other parts of the chloroplast in glaucophytes [11,12] (Figure 1A). To allow the outer envelope membrane to constrict, the PG layer at the division site has to be cut from the outermost site (a process called PG splitting), as in bacterial cell division. The PG splitting that takes place in glaucophyte chloroplast division should require PG hydrolyzing enzymes as is the case in bacterial cell division [14,15]. In addition, some algal and plant genomes encode homologs of cyanobacterial proteins that are involved in PG synthesis [16], although PGs have never been detected in chloroplasts other than glaucophytes. Inhibitors of PG synthesis [16-19] or disruption of the nucleus-encoded *mur* or *mra* genes [20] impairs chloroplast division in charophycean algae, the moss *P. patens*, and the lycophyte (fern) *Selaginella nipponica*. These observations raise the possibility that homologs of PG hydrolyzing proteins might also be related to chloroplast division in lineages other than glaucophytes. However, the relationship between PG splitting at the division site and the progression of chloroplast division has not been elucidated. Characterization of the PG dynamics in chloroplast division is important for an understanding of how the chloroplast division machinery has been modified from the original cyanobacterial division machinery by the eukaryotic host cell and how the ancestral algae regulated chloroplast division during the early stages of evolution. Recent studies of bacterial cell division using the Firmicutes (gram-positive) *Bacillus subtilis*[21] and Proteobacteria (gram-negative) *Escherichia coli*[22-24] and *Caulobacter crescentus*[25-27], have identified peptidase (DipM in *C. crescentus* and LytE in *B. subtilis*) or amidase (Ami in *E. coli*) and their activators (NlpD and EnvC in *E. coli*), all of which are involved in PG hydrolysis at the division site. In addition, the draft nuclear genome of the glaucophyte *C. paradoxa* was published recently [28]. Such information will facilitate a clarification of the above issue.

In this study, by surveying the *C. paradoxa* genome, it was confirmed that there are no known chloroplast division proteins of host eukaryotic origin in this alga. Instead, we found homologs of the DipM protein in cyanobacteria and *C. paradoxa*. These results suggest that PG hydrolysis by DipM participates in glaucophyte chloroplast division as it does in cyanobacterial cell division. In addition, we found that nucleus-encoded DipM homologs in charophycean algae and the moss *P. patens* are required for normal chloroplast division. Thus, the PG remodeling pathway is retained in the chloroplasts of Viridiplantae and plays some role in chloroplast division.

## Results

An earlier search in EST database of *C. paradoxa* failed to identify the dynamin-related protein DRP5B, glycogenin-like protein PDR1 (identified in the red alga *C. merolae*, it is a glucan filament of the outer PD ring) [3] and or the other known chloroplast division proteins that originated from eukaryotic host cell after the endosymbiotic event [13]. After the previous search efforts, the draft genome sequence of *C. paradoxa* was ultimately published [28] and thus we again searched the known chloroplast and cyanobacterial division proteins in the genome database. The search newly identified Ftn2/ARC6, FtsI, and MinC, which are descended from ancestral cyanobacteria (Figure 1B; Additional file 1: Table S1), whereas DRP5B, PDR1 and the other chloroplast division proteins of eukaryotic host origin were not present (Figure 1B). Thus, the known components of the chloroplast division machinery that function on the cytosolic side, in the outer envelope, or in the intermembrane space (the space between the outer and inner envelope membranes) in other lineages are missing in glaucophyte chloroplasts. Instead, *C. paradoxa* possess a protein homologous to FtsI, which localizes at the bacterial division site and cross-links the glycan strands, and a protein homologous to FtsW, which transports lipid-linked peptidoglycan precursors at the division site [29]. These observations led us to search *C. paradoxa* homologs of the proteins that are involved in PG splitting at the cyanobacterial division site.

Thus far, PG hydrolysis and its regulation at the bacterial division site have been studied in *B. subtilis*, *E. coli* and *C. crescentus*[14,15], but not in cyanobacterial species. The studies have shown that different sets of proteins are involved in PG hydrolysis at the division site in distinct bacterial lineages (Figure 1B). In *B. subtilis*, LytE, LytF and CwlS localize at the division site, where these proteins hydrolyze PG for cell separation [21]. These three proteins share a similar domain composition: the N-terminal portion consists of three to five repeats of the LysM motif, which binds the newly synthesized PG chains at the division site that have not been modified by teichoic acid [30]. The C-terminal portion contains the NlpC/P60 _D_,_L_-endopeptidase motif [21] (Figure 1B).

In *C. crescentus*, DipM is responsible for septal PG splitting. The N-terminal repeats of the LysM motif targets DipM to the septum and the C-terminal LytM/M23 domain hydrolyzes PG [25-27] (Figure 1B). *E coli* possesses a DipM homolog, NlpD, but NlpD does not hydrolyze PG. Instead, NlpD and another LytM domain-containing protein, EnvC, localize at the division site and activate the amidases, AmiA, AmiB, and AmiC, which then hydrolyze PG at the division site [22-24].

**Figure 2:**
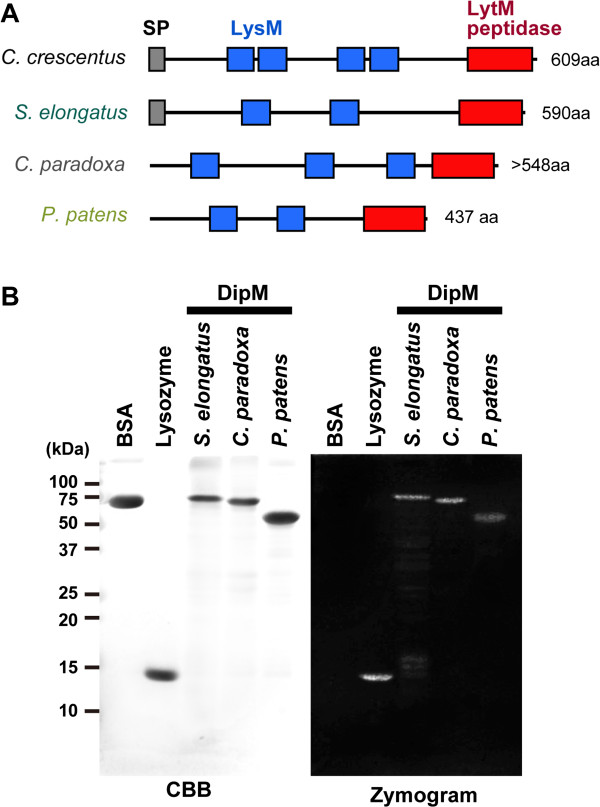
**DipM homologs of cyanobacteria and photosynthetic eukaryotes display PG hydrolase activity. (A)** Predicted domain structure of *C. crescentus* DipM and its homologs in the cyanobacterium *S. elongatus*, the glaucophyte alga *C. paradoxa* and the moss *P. patens*. SP, signal peptide. The GenBank accession numbers are summarized in Additional file 1: Table S1. For the *P. patens* DipM proteins, only DipM1 is shown. For *C. paradoxa* DipM, the deduced amino acid sequence lacks information on the N-terminal portion because we were unable to obtain full length cDNA. **(B)** Zymogram analysis of the PG hydrolase activity of the DipM homologs. *S. elongatus* (cyanobacterium), *C. paradoxa* (glaucophyte) and *P. patens* (moss) DipM homologs hydrolyzed murein sacculi in the gel (zymogram) which is indicated by negative staining with methylene blue. 5 μg of bovine serum albumin (BSA), lysozyme and recombinant DipM polypeptides were applied to SDS gels containing purified *S. elongatus* murein sacculi. The proteins in one of the gels were stained with Coomassie Brilliant Blue (CBB). The other gel was incubated in renaturation buffer and then areas of lysis were detected by staining of the murein sacculi with methylene blue. For the *P. patens* DipM proteins, only DipM1 was examined.

In order to both define which type of PG hydrolyzing mechanism is responsible for cyanobacterial cell division and address how the mechanism has been changed/lost in chloroplasts, we searched for homologs of the above mentioned proteins based on the existence of certain motifs in the respective cyanobacterial and eukaryotic genome databases. The search turned up homologs of the Ami amidases, DipM/NlpD and EnvC in cyanobacteria, although homologs of LtyE/LytF/CwlF were not found (Figure 1C and 2A; Additional file 1: Table S1). In photosynthetic eukaryotes, the search identified DipM homologs only in the glaucophyte *C. paradoxa* and the moss *P. patens* (Figure 1C and 2A; Additional file 1: Table S1). In addition, by searching the EST databases, we found DipM homologs (i.e. containing LysM and LytM domains) in charophycean algae, *Chaetosphaeridium globosum* (GI:372830473) and *Klebsormidium flaccidum* (GI:372624497), and the lycopodiophyte (a vascular plant) *Selaginella moellendorffii* (Figure 1C; Additional file 1: Table S1). All of these eukaryotic DipM homologs were most closely related to cyanobacterial DipM in the BLAST searches, suggesting that these eukaryotic proteins are descended from the original cyanobacterial endosymbiont. However we could not construct a reasonable alignment to conduct phylogenetic analyses, because sequence similarity was evident only in a limited region. Besides the above mentioned eukaryotic proteins of cyanobacterial origin, our search identified additional DipM homologs in *P. patens* which are closely related to proteins of bacteria other than cyanobacteria (GI:168061355; GI:168053993). The origin of the two additional proteins in *P. patens* is not clear, but it likely acquired by horizontal gene transfer from environmental bacteria.

### DipM in the photosynthetic eukaryotes *C. paradoxa* and *P. patens* displays PG hydrolase activity *in vitro*

To determine whether DipM homologs of *S. elongatus*, *C. paradoxa* and *P. patens* have the ability, like DipM, to hydrolyze PG in *C. crescentus*, the respective recombinant proteins (including both LysM and LytM domains) were produced in *E. coli* and tested for activity in a zymogram assay. To this end, *S. elongatus* DipM (recombinant protein without the signal peptide), as well as the control proteins bovine serum albumin (BSA) and lysozyme, were also applied to a denaturing SDS gel containing *S. elongatus* murein sacculi and refolded by removing the SDS from the gel. Subsequently, the gel was stained with a PG binding dye, which is supposed to produce clear zones in all areas in which the sacculi have been degraded by PG hydrolase activity (Figure 2B). Whereas BSA, as expected, was inactive in the assay, the DipM of *C. paradoxa* and *P. patens* produced a clear zone, as did *S. elongatus* DipM and lysozyme. These results indicate that the DipM forms of *C. paradoxa* and *P. patens* are able to hydrolyze PG *in vitro*.

### DipM localizes to the cell division site and is involved in cell division in the cyanobacterium *S. elongatus*

**Figure 3:**
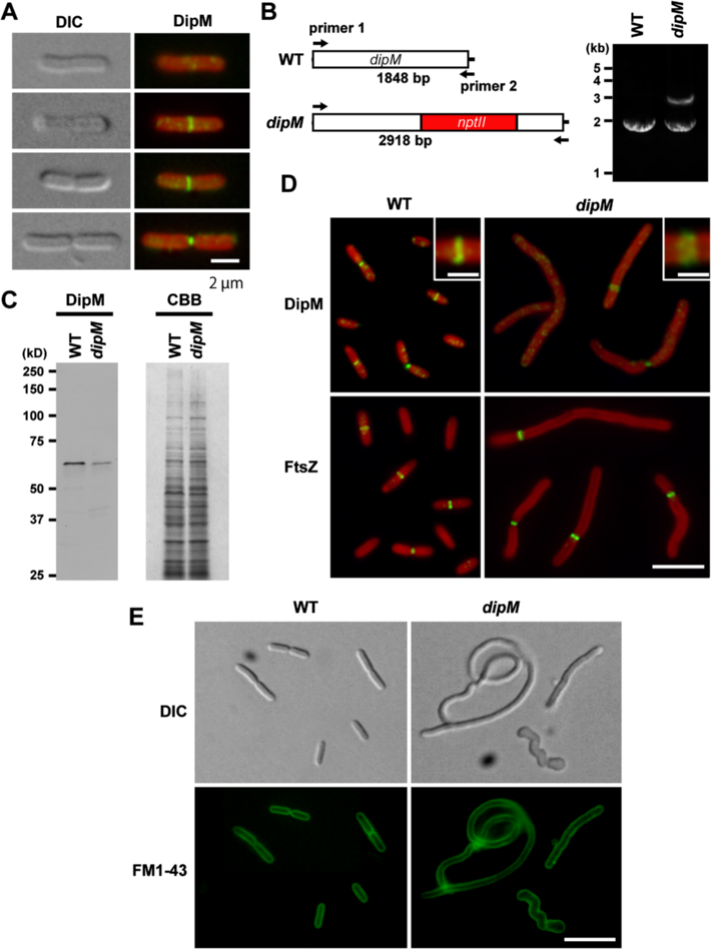
**DipM localizes at the mid cell position and is involved in cell division in the cyanobacterium *S. elongatus. *(A)** Immunofluorescent images showing DipM localization at the mid cell position (the green fluorescence) in *S. elongatus*. The red color is the autofluorescence of chlorophyll. Differential interference contrast (DIC) images of the same cell are also shown. **(B)** The mutated *S. elongatus dipM* locus. *nptII* gene was inserted into the *dipM* locus and the insertion was detected with PCR using primer 1 and primer 2. The PCR produced 2918-bp or 1848-bp products from the inserted and intact chromosomes, respectively. *S. elongatus* has multiple genomes and the amplification of the two bands in the mutant indicates that the mutant cell possesses both mutated and intact *dipM* loci. **(C)** Immunoblot analysis showing reduction of the DipM protein level in the *dipM* mutant. Equal amounts of total protein were loaded in each lane and the equality of loading was confirmed by Coomassie Brilliant Blue (CBB) staining after SDS-PAGE. **(D)** Analyses of FtsZ ring frequency and DipM localization in wild-type and *dipM* mutant cells. The *dipM* mutant cells display an elongated shape because of cell division defect and possesse a single FtsZ ring. Localization of DipM in the *dipM* mutant is relatively diffusive compared to the wild type. Magnified views of the DipM localization are shown in the insets. **(E)** FM1-43 staining of the wild type and *dipM* mutant showing the pattern in the cytoplasmic membrane. The *dipM* mutant cells have no membrane septa, suggesting that constriction of both the outer and cytoplasmic membranes is impaired. Scale bar = 2 μm **(A)**, 5 μm **(D)**, 1 μm (insets of **D**), 10 μm **(E)**.

Although the similarity in the primary structure suggests that the *S. elongatus* DipM is involved in cell division as are the *C. crescentus* DipM and *E. coli* NlpD, the function of this protein has not been characterized. In order to test the involvement of the *S. elongatus* DipM in cell division, we first examined the localization of DipM in *S. elongatus*. Immunofluorescence microscopy using antibodies against *S. elongatus* DipM showed that DipM localizes at the cell division site during both early and late constriction (Figure 3A) as were the case for DipM in *C. crescentus*[25-27] and NlpD in *E. coli*[22].

To examine whether DipM is required for cell division in *S. elongatus*, we tried to disrupt the *dipM* gene by replacing the locus with the *nptII* gene, which confers kanamycin resistance. Because cyanobacteria have multiple genomes [31], PCR was used to determine whether the mutation was completely or incompletely segregated. Even after five serial transfers of single kanamycin-resistant colonies to new plates, we were unable to completely segregate the *dipM* disruptant (Figure 3B), suggesting that complete depletion of the DipM protein is lethal in *S. elongatus*. Immunoblot analysis showed that the DipM level is reduced in the incompletely segregated *dipM* mutant compared to the wild type (Figure 3C). The *dipM* mutant cells displayed an elongated shape indicative of a cell division defect. In addition, the mutant cells are wider than the wild-type cells and some mutant cells exhibited a twisted shape (Figures 3D and 3E) probably because remodeling of PG layer is impaired in the *dipM* mutant. Similar heterogenous appearance was also observed in Δ*dipM* mutant of *C. crescentus*[26]. Since the incompletely-segregated *dipM* mutant cell still produced a certain level of the DipM protein and was still able to divide (doubling time of *dipM* mutant was similar to that of the wild type), we examined DipM localization in the *dipM* mutant. Immunofluorescence microscopy showed that DipM localizes at the presumed division site, but the fluorescent signal is lower than in the wild-type and the localization is relatively diffusive (Figure 3D). Thus, in the *dipM* mutant, DipM localization and concentration at the division site is impaired, probably because of a shortage of the DipM protein.

FM1-43 (a membrane dye) staining [22] showed that elongated *S. elongatus dipM* mutant cells have no membrane septa (Figure 3E), suggesting that constriction of both the outer and cytoplasmic membranes is impaired in the mutant cells. Consistent with this observation, the elongated *S. elongatus dipM* mutant cell usually had a single FtsZ ring (Figure 3D).

### DipM localizes to the site of cell division and is involved in cell division in the cyanobacterium *S. elongatus*

**Figure 4:**
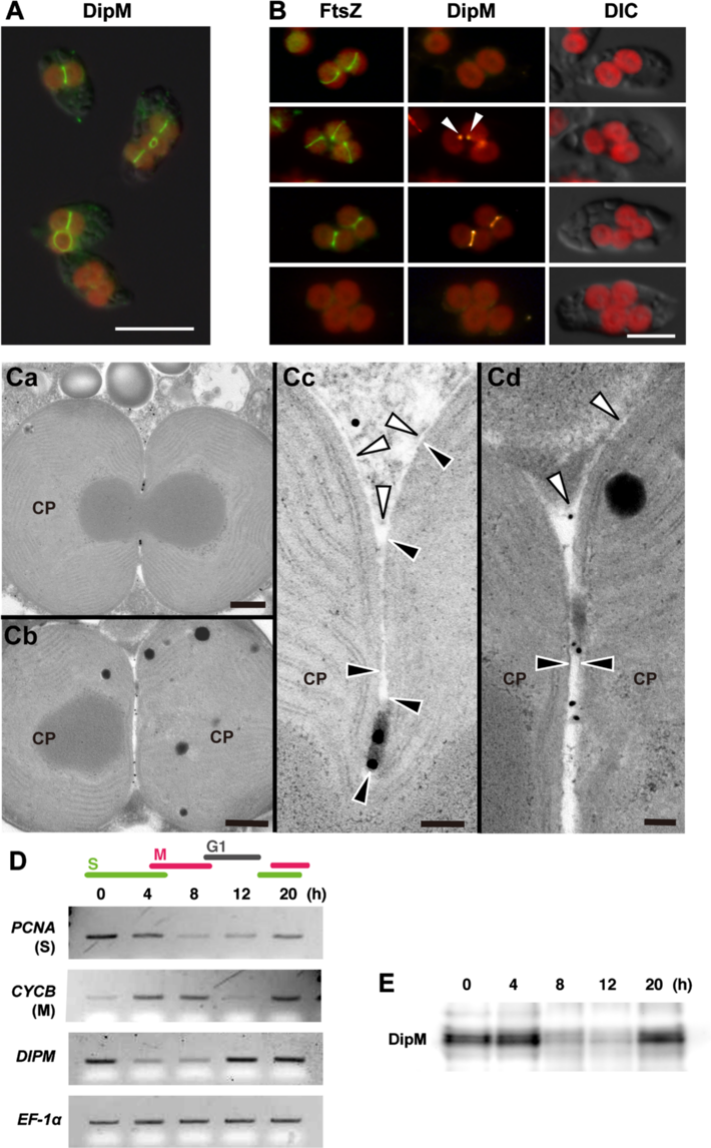
**DipM is preferentially expressed in the S phase and localizes at the chloroplast division site in the intermembrane space in the glaucophyte alga *C. paradoxa. *(A)** Immunofluorescent images showing DipM localization at the chloroplast division site (the green fluorescence) in *C. paradoxa*. The red color is the autofluorescence of chlorophyll. **(B)** Immunofluorescent images showing FtsZ (the green fluorescence) and DipM (the yellow fluorescence) localization in *C. paradoxa*. DipM localizes at the division site after FtsZ ring formation. **(C)** Immunoelectron micrographs showing the localization of DipM at the PG layer at the chloroplast division site. The gold particles indicate the location of DipM. The white and black arrowheads indicate the outer and the inner envelope membranes of the chloroplast (CP), respectively. **(D)** Semiquantitative RT–PCR analyses showing the change in the mRNA level of *DIPM* during synchronous culture. *DIPM* mRNA level peaks during the S phase. The chloroplast division genes are boxed. *EF*-*1*α was used as the quantitative control. *PCNA* and *CYCB* were used as the S- and M-phase markers. Cells were arrested in the S phase with aphidicolin (an inhibitor of DNA polymerase) and then the cell cycle was restarted under continuous light along with the removal of aphidicolin, as described (Miyagishima et al., [32]). **(E)** Immunoblot analysis showing the change in the level of the DipM protein during synchronous culture. DipM protein level peaks during the S phase. Equal amounts of total protein were loaded in each lane. Scale bar = 10 μm **(A)**, 5 μm **(B)**, 500 nm (Ca and Cb), 100 nm (Cc and Cd).

The above results confirmed the involvement of DipM in cyanobacterial cell division. We then asked whether nucleus-encoded DipM is associated with chloroplast division in the glaucophyte *C. paradoxa*. Bacterial DipM possesses the signal peptide and is secreted into the periplasm where it binds/hydrolyzes PG [25-27]. Thus, *C. paradoxa* DipM likely localizes at the chloroplast division site in the intermembrane space where the PG layer exists. Immunofluorescence microscopy using an anti-*C. paradoxa* DipM antibody showed that DipM localizes at the chloroplast division site in *C. paradoxa* (Figure 4A). Simultaneous immunostaining of FtsZ and DipM showed that DipM localizes at the division site after FtsZ ring formation and before the division site constriction (Figure 4B). We frequently observed a single spot of DipM staining (Figure 4B, arrowheads) colocalized with a portion of an FtsZ ring at the expected division site (Figure 4B). This observation suggests that DipM localization, and possibly PG hydrolysis, starts from a specific point and extends across the entire span of the division site. Immunoelectron microscopy showed that DipM (detected by gold particles) localizes at the PG layer in the intermembrane space at the division site (Figure 4C). DipM localizes at the PG layer even after inner envelope division (Figures 4Cb and 4Cd), i.e. when constriction of the outer envelope is still in progress. *C. paradoxa* is not genetically tractable, at present, and molecular genetic analyses of DipM function are therefore not feasible. However, the findings of DipM localization and PG hydrolyzing activity suggest that nucleus-encoded DipM is imported into the intermembrane space at the division site, where it is involved in PG degradation.

In a previous study, we showed that the transcript and protein levels of nucleus-encoded *MIND* and *MINE*, but not those of *FTSZ*, change through the course of cell cycle progression, peaking during the S phase when chloroplasts divide [32]. In order to examine whether *DIPM* transcription and translation are also regulated by the cell cycle, *C. paradoxa* was synchronized by arresting the cells in the S phase with aphidicolin and restarting the cell cycle by removing aphidicolin. Semiquantitative RT-PCR analyses of an S-phase marker, *PCNA*, and an M-phase marker, *CYCB* (encoding an M-phase cyclin), showed that the culture was synchronized (Figure 4D). The RT-PCR (Figure 4D) and immunoblot analyses (Figure 4E) showed that the *DIPM* transcript and DipM protein levels change through the cell cycle progression and peak during the S phase, as do *MIND* and *MINE*[32]. Thus, DipM transcription and translation are regulated by the cell cycle such that DipM is specifically expressed during chloroplast division.

### DipM is required for normal chloroplast division in the moss *P. patens*

These results suggest a conservation of DipM function between cyanobacteria and glaucophyte chloroplasts in which DipM probably hydrolyzes PG at the division site. This conservation is reasonable, because glaucophyte chloroplasts retain a PG layer that is descended from the ancestral endosymbiotic cyanobacterium. No PG layers have been detected in any chloroplasts (plastids) other than glaucophytes. However, our database search identified DipM homologs in charophytes, the moss *P. patens* and the fern *S. moellendorffii*. Interestingly, these lineages still retain the genes for PG synthesis, which are probably functional in chloroplasts, despite of there being no evidence for the existence of PG. In addition, mutations in these genes as well as certain inhibitors of PG synthesis impair chloroplast division [16].

**Figure 5:**
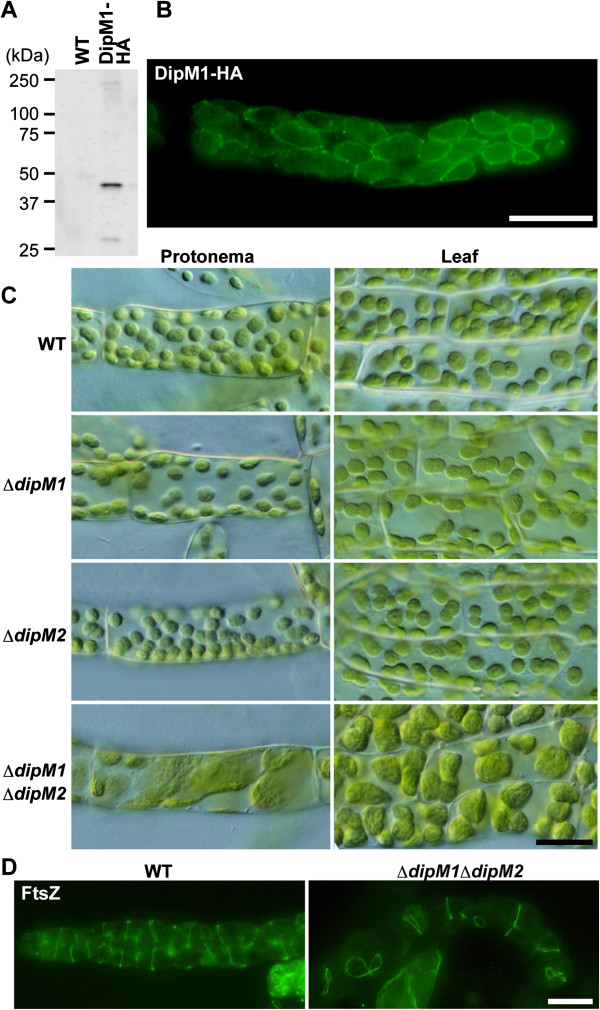
**DipM is required for chloroplast division in the moss *P. patens. *(A)** An immunoblot analysis showing the expression of the DipM1-HA fusion protein. DipM1-HA was expressed by the rice actin promoter and was detected by an anti-HA antibody. **(B)** An immunofluorescent image showing DipM1-HA (the green fluorescence) localization over the entire surface of the chloroplast. **(C)** Phenotypes of ∆*dipM1*, ∆*dipM2* as well as ∆*dipM1* and ∆*dipM2* double mutants. The ∆*dipM1* and ∆*dipM2* double mutant cells contain a smaller number of the larger chloroplasts than the wild type, which is indicative of a chloroplast division defect. Chloroplasts in protonema and leaf cells of the wild type (WT) and mutants were observed by differential interference contrast microscopy. **(D)** Immunofluorescent images showing FtsZ localization in the wild-type (WT) and ∆*dipM1* ∆*dipM2* protonemal cells. Most of the chloroplasts in ∆*dipM1* ∆*dipM2* have a single FtsZ ring, suggesting that DipM1 and DipM2 are required for chloroplast division after FtsZ ring formation. Scale bar = 20 μm **(B, C and D)**.

In order to examine DipM function in land plants, we examined localization and the effects of *dipM* mutation on chloroplast division in the moss *P. patens*. Despite several efforts, we were unable to obtain antibodies specifically reacting with *P. patens* DipM1. Therefore, we expressed DipM1-HA using an actin-promoter in *P. patens* and examined the localization of DipM1-HA with an anti-HA antibody. Immunoblot analysis showed there was expression of DipM1-HA in the transgenic *P. patens* (Figure 5A). However, the size of the detected band (~45 kDa) was smaller than the expected size of DipM1-HA (~52 kDa) (Figure 5A), suggesting that the N-terminal portion is processed *in vivo*, despite the computer prediction of there being no N-terminal signal or transit peptide. Immunofluorescence microscopy using the HA-antibody showed that DipM1-HA localizes at the surface of chloroplasts (Figure 5B). Based on the DipM localization in bacteria and *C. paradoxa*, DipM1-HA probably exists in the intermembrane space. However, in contrast to *S. elongatus* (Figure 3) and *C. paradoxa*, DipM1-HA (Figure 4) localizes over the entire surface of the chloroplast and we did not observe any ring-like localization or localization specific to the chloroplast division site in *P. patens* (Figure 5B).

In order to investigate whether DipM is involved in chloroplast division in *P. patens*, like the proteins related to PG synthesis [20], we disrupted the *DipM1* and *DipM2* loci of *P. patens* (Additional file 2: Figure S1). There were no detectable differences in chloroplast size or shape in both the protonemal and leaf cells between the wild type and Δ*dipM1* or Δ*dipM2* mutant (Figure 5C). However, the Δ*dipM1* and Δ*dipM2* double mutant cells contained a smaller number of the larger chloroplasts than the wild-type cells (Figure 5C). This phenotype is indicative of a chloroplast division defect and similar to that observed in *P. patens* mutants in which genes homologous to bacterial PG synthesis genes are inactivated [20]. These observations suggest that DipM1 and DipM2 play a redundant role in chloroplast division, probably in the intermembrane space in the case of *P. patens*. In order to define the stages of the chloroplast division process for which DipM1 and DipM2 are required, we then examined FtsZ localization in the Δ*dipM1* Δ*dipM2* double mutant. Immunofluorescence microscopy using anti-*A. thaliana* FtsZ2-1 antibodies showed that FtsZ forms a ring structure in the enlarged chloroplast (Figure 5D). In our findings, most of the chloroplasts have a single FtsZ ring. This situation is similar to the FtsZ localization pattern in the cyanobacterium *S. elongatus dipM* mutant. This FtsZ localization pattern in the Δ*dipM1* Δ*dipM2* mutant suggests that DipM1 and DipM2 are required after FtsZ ring formation, as is the case for bacterial DipM/NlpD [22,25-27].

## Discussion

Recent molecular genetic and structural studies have identified proteins that are involved in chloroplast division and provided insight into how the chloroplast division machinery has been modified from the cyanobacterial division machinery during the course of evolution. These studies have revealed the retention of certain cyanobacterial division machinery components inside the chloroplasts, and the provision of additional machinery by the eukaryotic host cell both on the inside and outside of the chloroplasts [2-6]. However, none of the components of eukaryotic host origin, which function on the cytosolic side of the chloroplast division site, are evident in glaucophyte algae. In glaucophytes, PG ingrowth and PG splitting in the intermembrane space at the division site is accompanied by chloroplast division, unlike in any of the other lineages. Thus, in order to understand how the chloroplast division machinery was remodeled in the early stages of evolution, it is important to understand how the PG layer and the attached outer envelope membrane are separated in glaucophyte chloroplast division.

### Comparison of PG splitting in Proteobacteria, Firmicutes and Cyanobacteria

Although less well characterized compared to the FtsZ-based division complex on the cytosolic side, recent molecular genetic studies have started to elucidate the molecular mechanisms of PG splitting during bacterial cell division [14,15,21-27]. In bacterial cell division, the site of division is established by localized polymerization of the tubulin-like FtsZ GTPase into a structure that acts as a scaffold for the assembly of other “divisome” components. One of the primary functions of the divisome is to promote the synthesis of the PG layer that will eventually fortify the new daughter cell poles. This involves several divisome-associated PBPs (penicillin-binding proteins) [14,15]. Although the septal PG produced by these synthases is initially shared by the daughter cells, it must be split after it is formed to allow constriction of the outer membrane (in the case of gram negative bacteria) and the final split of the daughter cells. Just as there are differences in the composition of cytoplasmic division complex between gram-negative and gram-positive bacteria [14,15], there is a notable difference in the structure of the PG layer and septal PG splitting between them. In gram-positive bacteria, this septal PG layer is typically split sometime after the daughter cells have been compartmentalized by membrane fusion. In gram-negative bacteria, however, the septal PG layer is split shortly after it is formed in order to allow constriction of the outer membrane to closely follow that of the inner (cytoplasmic) membrane [14,15]. In this regard, electron microscopy has shown that cyanobacterial cell division and glaucophyte chloroplast division is more like the cell division of Firmicutes rather than Proteobacteria in terms of PG ingrowth and splitting, although cyanobacteria do have the outer membrane characteristic of Gram-negative bacteria [11,12] (Figure 4C). This similarity in PG splitting between Firmicutes and cyanobacteria is likely related to the Gram-positive-like characteristic of the PG layer in cyanobacteria [33].

Despite the similarity between cyanobacteria and Firmicutes, LytE homologs (i.e. proteins containing both LysM and NlpC motifs) are not evident in the cyanobacterial genome and instead our database searches identified DipM and EnvC homologs in the cyanobacterial genome (Figure 1). In *S. elongatus*, it was not possible to completely deplete DipM (Figure 3), probably because complete depletion is lethal. The mutant cell, in which DipM is downregulated, exhibited an elongated shape because of cell division defect and usually possessed a single FtsZ ring (Figure 3). These results are in contrast to those in *E. coli*, in which deletion of NlpD alone causes no apparent cell division defects. When PG splitting is impaired by deletion of NlpD and EnvC or sets of Ami proteins, *E coli* cells form chains that are connected by PG septa at fairly regular intervals (i.e. cytoplasmic membrane fission occurs without PG splitting) [22-24]. However, in a manner similar to the *S. elongatus dipM* mutant (Figure 3), *C. crescentus dipM* mutation leads to filamentation of cells that fail to invaginate both the cytoplasmic and outer membrane [25-27]. Presumably, in both *C. crescentus* and *S. elongatus*, the constriction of the FtsZ ring and the cytoplasmic membrane are tightly coordinated with PG splitting, in which the down regulation of DipM activity also delays constriction of the cytoplasmic membrane.

### PG splitting in glaucophyte chloroplasts

In chloroplast division in lineages other than glaucophytes, the outer PD ring and the dynamin-related protein DRP5B are believed to produce the motive force from the cytosolic side to constrict the outer envelope membrane [2-6]. In contrast, these elements are missing in glaucophytes, and instead invagination of the outer envelope membrane appears to be a passive process, as tethering the membrane to the PG layer causes the outer envelope membrane to move inwards as the septal PG is split during constriction. In Proteobacteria, abundant murein-binding outer membrane proteins, such as Lpp and OmpA bind the outer membrane to the PG. In addition, recent studies have shown that Pal, an abundant outer membrane lipoprotein, localizes to the division site, and interaction of Pal and the inner membrane protein Tol is required for tethering the outer membrane to PG during cell division [14,15,34]. However, homologs of these outer membrane proteins are not present in the cyanobacterial and glaucophyte genomes except for proteins partially similar to Lpp and OmpA in a limited number of species of cyanobacteria. Thus, further investigations using cyanobacteria are needed to understand how the outer membrane or outer envelope constriction is coupled with PG splitting in both cyanobacteria and glaucophyte chloroplasts.

Our findings showed that DipM localizes at the chloroplast division site after FtsZ ring formation and that the DipM localization starts from a specific point and then moves the entire span of the division site (Figure 4B). This pattern of DipM localization is consistent with previous observation by electron microscopy which showed that invagination of the outer envelope starts from a specific point and spreads over the entire of the division site [12]. Given that *C. paradoxa* DipM displays PG hydrolyzing activity *in vitro* (Figure 2), PG splitting starts from a specific point and then proceeds to the entire division site in *C. paradoxa*.

In *C. paradoxa*, DipM is preferentially expressed in the S phase (Figure 4D and 4E). Our previous study showed that FtsZ is persistently expressed throughout the cell cycle, whereas the expression of the nucleus-encoded MinD and MinE, as well as FtsZ ring formation, are restricted to the S phase [32]. In contrast to the nucleus-encoded chloroplast division genes, the expression of the chloroplast-encoded division genes (*ftsW* and *sepF*, Figure 1C) is not regulated by the host cell cycle [32]. These results suggest that cell-cycle-based transcriptional/translational regulation of some, but not all, chloroplast division genes is responsible for the synchronization of chloroplast division and the host cell cycle. Given that chloroplast division proteins of the host eukaryotic origin are not present in *C. paradoxa*, endosymbiotic gene transfer and establishment of transcriptional/translational regulation likely occurred earlier than the addition of the division proteins of eukaryotic host origin, such as DRP5B and PDR1. Given that cell division but not the growth of *S. elongatus* requires DipM (Figure 3), endosymbiotic gene transfer and coupling of the timing of DipM expression to the host cell cycle appear to be sufficient to synchronize the timing of endosymbiont cell division with the host cell cycle. Numerous eukaryotic species contain bacterial or eukaryotic endosymbionts other than mitochondria and chloroplasts. In most cases, endosymbiotic bacteria retain a PG layer and in some cases, such as cyanobacterial endosymbionts in the cercozoa *Paulinella chromatophora*[35] and the diatom *Rhopalodia gibba*[36], the timing of the cell division of the endosymbionts is tightly coupled to the host cell cycle. Therefore, an understanding the regulation of chloroplast division, in which PG splitting is involved, will shed light on the common features which underlie the establishment and further evolution of permanent endosymbiotic relationships.

### Phylogenetic distribution of PG synthesis and splitting proteins, and the involvement of these proteins in chloroplast division

In the database searches we performed, DipM homologs were also found in charophytes, mosses and a lycophyte (fern), but not in red algae, green algae or seed plants (Figure 1). In addition, our results suggest that DipM is required for chloroplast division after FtsZ ring formation in the moss *P. patens* (Figure 5). The phylogenetic distribution of DipM is consistent with that of proteins homologous to enzymes that are involved in PG synthesis in bacteria and in the impairment of chloroplast division by PG synthesis inhibitors [16,19]. This agreement and the PG hydrolase activity of DipM homologs *in vitro* (Figure 2) suggest that some lineages of Viridiplantae still possess a PG layer in the intermembrane space of chloroplasts. Although PG has never been detected in chloroplasts other than glaucophyte, analyses of a greater sensitivity will be required to ultimately address this issue. In *P. patens*, DipM localization is not restricted to the division site (Figure 5B). Thus, PG would exist in entire intermembrane space in *P. patens*, if it exists. As suggested previously regarding PG synthesis, the phylogenetic distribution of DipM indicates that PG hydrolyzing enzymes were also independently lost in distinct lineages at least three times from ancestral red algae, chlorophytes and seed plants (Figure 1).

## Conclusions

As is the case for cyanobacterial cell division, chloroplast division in the glaucophyte *C. paradoxa* involves PG splitting rather than constriction by the outer PD ring and the dynamin-related protein. The PG splitting is mediated by DipM protein and this activity is likely still involved in chloroplast division in Viridiplantae.

## Methods

### Database search

DNA and protein sequence databases were accessed at the National Center for Biotechnology Information (http://www.ncbi.nlm.nih.gov) and from the Cyanophora Genome Project (http://cyanophora.rutgers.edu/cyanophora/home.php). Eukaryotic homologs of the known bacterial cell division genes were identified based on amino acid sequences using the Basic Local Alignment Search Tool (TBLASTN and BLASTP) [37]. Protein motifs were searched by PFAM (http://www.sanger.ac.uk/Software/Pfam/).

### Culture conditions

*S. elongatus* PCC 7942 and its derivatives were grown in BG-11 medium at 30˚C under continuous light (30 μmol photons m^−2^ s^−1^). *C. paradoxa* UTEX555 (NIES-547) was grown in C medium (http://mcc.nies.go.jp/02medium-e.html) at 24 °C under continuous light (30 μmol photons m^−2^ s^−1^). *P. patens* subsp. *patens* and its derivatives were grown at 20°C on the minimal medium (BCD medium) supplemented with 5 mM diammonium (+)-tartrate (BCDAT) or supplemented with 0.5% glucose (BCDG medium) (http://www.plant-biotech.net/) under constant light (50 μmol photons m^−2^ s^−1^) as described [38].

### Preparation of recombinant DipM proteins and antibodies

The cDNA sequence encoding the full length or a partial fragment of the respective protein was amplified by PCR using the primers 5’-caccGGCAAACTGACCAAGTTCAGAT-3’ and 5’-CTAGCGAGAAGGGAGATAGGCGAT-3’ for *S. elongatus* DipM, 5’-caccGAGGAGCTCTTTTCAACTCCG-3’ and 5’-TCAGCACCGCATGTCGAGGTAG-3’ for *C. paradoxa* DipM, and 5’-caccGTGTTCAAGTGGCCGACTCTAAGG-3’ and 5’-TCACAAACGGACCCATTTCAAT-3’ for *P. patens* DipM1. These PCR products were cloned into a pET100 expression vector (Invitrogen) and 6xHis fusion polypeptides were expressed in Rosetta (DE3) *Escherichia coli* cells and purified using a HisTrap HP column (GE healthcare). The antisera against *S. elongatus* and *C. paradoxa* DipM were raised in rabbits using the respective recombinant polypeptides. Antibodies were affinity-purified from the respective antisera by using the respective recombinant proteins coupled to a HiTrap NHS-activated HP column (GE Healthcare).

### Zymography

Zymography was performed essentially as described elsewhere [25], except that the cell wall was prepared from *S. elongatus*. For cell wall preparation, cells (1 L, OD730 = 1.0) were harvested by centrifugation at 4,000 *g* for 15 min. Cells were resuspended in 10 mL of 5% SDS and then sonicated 50 times with a duty cycle of 10 s on and 10 s off on ice. The SDS-insoluble fraction was harvested by centrifugation at 15,000 *g* for 20 min. The pellet was resuspended in 10 mL of 50 mM Tris–HCl, pH 7.5 containing 2 mg/mL Pronase (Roche) and incubated at 37˚C overnight. The resultant cell wall was washed with 0.1% SDS by centrifugation at 15,000 *g* for 20 min three times. The washed cell wall (~100 mg, wet weight) was suspended in 1 mL of distilled water.

5 μg each of DipM polypeptides that were purified by a HisTrap column as described above, lysozyme and bovine serum albumin were separated by two SDS-PAGE gels (15%T) containing 0.6% (wet w/v) of the cell wall that were run concurrently. One gel was fixed and stained with Coomassie Brilliant Blue. The other was incubated overnight in renaturation buffer (25 mM Tris–HCl, pH 8.0, 1% TritonX-100), stained with 0.1% methylene blue in 0.01% KOH for 3 h and destained with distilled water.

### Synchronous culture and semi-quantitative RT-PCR of *C. paradoxa*

Synchronization and semi-quantitative PCR were performed as described [32]. *C. paradoxa* cells were cultured to a cell density of 1 × 10^6^ cells/mL at 24 °C under continuous light (40 μmol photons m^−2^ s^−1^) and aeration with ordinary air. To arrest the cells in the S phase, a 1/1,000 volume of 5 mg/ml aphidicolin solution in DMSO was added to the culture and cells were cultured for 24 h. To remove aphidicolin, cells were washed twice with fresh medium by centrifugation at 200 *g* for 10 min and then cultured under the same conditions as above.

For RT-PCR, total RNA was extracted from 5 mL culture with TRIzol reagent (Invitrogen). After DNaseI-treatment, cDNA was synthesized from the RNA using a random hexamer with ThermoScript RT (Invitrogen) and was treated with DNaseI. The PCR reactions were performed using the primers 5’-CGAGCACCTTGGGATTCCAGAG-3’ and 5’-GCTTGTTGCCTTGGTGAAGTTG-3’ for *PCNA*, 5’-AGGACAAACGCCACATGAACCC-3’ and 5’-TACGAGGACTCCACGCCAGCC-3’ for *CYCB*, 5’-CCCCACAGCCTGAACAACTTC-3’ and 5’-GAACGATGAGGACGTTGACAG-3’ for *DIPM*, and 5’-GGCTACAACCCCGACAAGATTC-3’ and 5’-CACGGCGGATGTCCTTGACG-3’ for *EF*-*1*α.

### Gene disruption of *S. elongatus*

To inactivate the *S. elongatus dipM* gene, the relevant genomic region (~1.7 kbp) was amplified with the primers 5’-CCATTCATCGACTGTCGCAGTT-3’ and 5’-AGAAGGGAGATAGGCGATCGGG-3’. The amplified DNA was cloned into the pGEM-T easy vector (Promega). The kanamycin resistance gene was amplified from a pUC4K vector (GenBank accession number × 06404) by the primers 5’-TGTGGAATTGTGAGCGGATAAC-3’ and 5’-AAGTCAGCGTAATGCTCTGCCA-3’. The amplified kanamycin resistance gene was inserted into the *Nru*I site of *dipM*. A construct in which the kanamycin resistance gene was inserted in the same orientation as the *dipM* gene was used for gene disruption. The *dipM* disruptant was generated by transformation of wild-type cells with the construct and selected on BG-11 plates containing kanamycin (15 μg ml^−1^). The single colonies were streaked on new plates five times. Segregation of the mutations was examined by PCR using the primers 5’-TAGGTAGTTTGTGGCGAATGGG-3’ and 5’-CCTCTCAACACGTAAAAGCGAT-3’.

### Gene disruption and expression of HA-tagged protein in *P. patens*

To inactivate *P. patens DipM1* and *DipM2*, the respective genomic region was amplified by the primers 5’-CATATCGTTCACTGAGCAGCGTC-3’ and 5’-ATTGGTAGAGTTGGGCTGGCTTC-3’ for *DipM1* and 5’-GTTCAAGTGGTTGACTCCCAAGC-3’ and 5’-CAGAACACCTTGCACGCTAGAGA-3’ for *DipM2*, respectively. The amplified DNA was cloned into pGEM-T easy. The kanamycin-resistance gene was cut from the pTN81 vector by *Eco*RV and inserted into the *Msc*I site of *DipM1*. The hygromycin-resistance gene was amplified from the 9WH3 vector by the primers 5’-AATGCTAACCCACAGATGGT-3’ and 5’-ATGGCTCTGATACCAATTTTTAAG-3’ and inserted into the *Nru*I site of *DipM2*.

The resultant *DipM1* (kanamycin-resistance) and *DipM2* (hygromycin-resistance) inactivation cassettes were cut from pGEM-T easy by *Not*I and introduced into the wild-type *P. patens* protonemal cells by particle bombardment, as described previously [39]. Transformed mosses were cultured on BCDG medium under darkness for 3 days and then transferred to BCDAT medium containing 50 μg ml^−1^ G418 or 30 μg ml^−1^ hygromycin for 2 weeks. The selected mosses were transferred onto BCDAT medium and allowed to grow for 1 week. Then they were transferred again onto the selection medium. *DipM1*and *DipM2* disruption were confirmed by PCR with the primers 5’-tcaagcatcagcttacaagtggca-3’ and 5’-atatctagttacaaaccctccttca-3’ for *DipM1*, and the primers 5’-cctacactgggatgctggctctaa-3’ and 5’-gcagccactttcgctaggtattga-3’ for *DipM2*, respectively (Additional file 2: Figure S1). To generate the *dipM1dipM2* double mutant, the *DipM2* gene in the *dipM1* disruptant was disrupted (Additional file 2: Figure S1).

To express the C-terminal HA epitope fusion of DipM1, *DipM1* cDNA was amplified by the primers atggtgttcaagtggccgactc and ttacgcgtaatctggaacgtcataagggtatcctgcatagtccgggacgtcatagggatagcccgcatagtcaggaacatcgtatgggtacaaacggacccatttcaatggg (the stop codon and 3xHA are underlined) and inserted into the *Swa*I site of a 9W3H vector, which drives the inserted gene by the rice actin promoter. The resultant vector was digested by *Not*I and was introduced into the wild type *P. patens*. Transformants were selected on BCDAT medium containing 30 μg ml^−1^ hygromycin.

### Immunofluorescence and immunoelectron microscopy

Immunofluorescence detection of DipM and FtsZ in *S. elongatus* and *C. paradoxa* was performed essentially as described [32,40]. Cells were fixed in 3% (w/v) paraformaldehyde dissolved in 50 mM PIPES-KOH, pH 6.8, 10 mM EGTA, 5 mM MgSO_4_ for 30 min at room temperature and washed twice with PBS-T (0.01% Tween-20 in PBS). After treatment with 0.05% Triton X-100 in PBS-T for 15 min, the samples were permeabilized for 30 min at 37°C with 0.2 mg ml^−1^ lysozyme dissolved in Tris–HCl, pH 7.5, 10 mM EDTA, and then washed twice with PBS. Following blocking with 2% bovine serum albumin in PBS-T (blocking buffer) for 30 min, cells were labeled at 30°C for 2 h with the first antibodies diluted in blocking buffer. Cells were then washed twice with blocking buffer, and incubated with Alexa Fluor 488 goat anti-rabbit IgG (H + L) (Invitrogen) diluted in the blocking buffer at a concentration of 1:1,000 at room temperature for 1 h. After washing twice with PBS-T, cells were observed by fluorescence microscopy (BX-51; Olympus). For simultaneous detection of DipM and FtsZ in *C. paradoxa*, the primary antibodies were directly labeled with fluorescent probes by using Mix-n-Stain CF Dye Antibody labeling kits (Biotium; CF555 for DipM antibodies and CF488 for FtsZ antibodies). Antibodies against *S. elongatus* DipM (1:500), *Anabaena* PCC 7120 FtsZ (1:500 to detect *S. elongatus* FtsZ; Agrisera), *C. paradoxa* FtsZ [32] (1:400), and *C. paradoxa* DipM (1:500) were diluted as indicated. Immunofluorescence detection of DipM1-HA and FtsZ in *P. patens* was performed according to PHYSCObase (http://moss.nibb.ac.jp/). Antibodies against *A. thaliana* FtsZ2-1 [41] (1:400) and the anti-HA antibody (1:100; Roche, 3 F10) were diluted as indicated.

For immunoelectron microscopy for the detection of DipM in *C. paradoxa* by a pre-embedding labeling method, cells were fixed in 4% paraformaldehyde and 0.2% glutaraldehyde dissolved in 0.1 M phosphate buffer, pH 7.4 for 1 h at 4˚C, and washed three times with PBS-T. After washing with PBS-T three times, cells were permeabilized by Triton X-100 and lysozyme as described above. After blocking with the blocking buffer, cells were reacted with the anti-*C. paradoxa* DipM antibodies (1:100 in the blocking buffer) for 4 h at room temperature. After washing with PBS-T four times, cells were reacted with 1.4-nm gold particle-conjugated secondary antibody (1:80 in blocking buffer) at 4˚C overnight. After washing with PBS-T four times, cells were post-fixed with 2% glutaraldehyde in the phosphate buffer at 4˚C overnight. Then the gold particles were enhanced with GOLD ENHANCE EM Formulation (Nanoprobes) according to the manufacturer’s instructions followed by post fixation with 2% osmium tetroxide in the phosphate buffer at 4˚C for 1 h. The cells were dehydrated and embedded in Quetol-812. Thin sections (90 nm thick) were stained with uranium and lead, and were observed under transmission electron microscope (JEM-1400Plus; JEOL).

### Immunoblotting

Cells were disrupted by sonication in 20 mM Tris–HCl, pH7.5, 8 M urea, 0.1% Triton X-100 and Complete Mini protease inhibitor mixture (Roche). After disruption, the samples were centrifuged at 15,000 *g* for 10 min and the supernatant fraction was used for immunoblotting. The protein content in the supernatant fraction was determined by Bradford assay (Bio-Rad). SDS-PAGE and Immunoblot analyses were performed as previously described [41]. The primary antibody against *S. elongatus* DipM (1:1,000), *C. paradoxa* DipM (1:1,000) and the HA epitope (Roche, 3 F10, 1:1,000) were diluted as indicated. The primary antibodies were detected by horseradish peroxidase–conjugated goat anti-rabbit or anti-rat antibody. The signal was detected with the ECL Prime Western Blotting Detection System (GE Healthcare) and the VersaDoc 5000 imaging system (Bio-Rad).

## Competing interests

The authors declare that they have no competing interest.

## Authors’ contributions

SM designed the study. SM and TF performed experiments using *C. paradoxa*. YK, CS and MS performed experiments using *P. patens*. SM performed all other experiments. SM wrote the manuscript. After modifications by all authors, all authors approved the final manuscript.

## Supplementary Material

Additional file 1: Table S1 GenInfo Identifier (GI) numbers or locus IDs of the amino acid or nucleotide sequences of cyanobacterial and chloroplast division proteins. Click here for file

Additional file 2: Figure S1 Confirmation of *DipM1* and *DipM2* disruption in *P. patens*. (A) Insertional mutation of the *P. patens DipM1* locus. The *nptII* gene was inserted into the *DipM1* locus and the insertion was detected by PCR. The PCR resulted in 3.9-kbp or 1.9-kbp products from inserted or intact chromosomes, respectively. #3, #8, and #11 were used for further analyses. (B) Insertional mutation of *P. patens DipM2* locus. *hpt* gene was inserted into *DipM2* locus and the insertion was detected by PCR. The PCR produces 3.2-kbp or 1.2-kbp products from inserted or intact chromosomes, respectively. #1 and #10 were used for further analyses. (C) *hpt* gene was inserted into *DipM2* locus of ∆*DipM1* mutant. The insertion was checked by PCR as in (B). #68 and #82 were used for further analyses. Click here for file
