# Supplementary material for: DipM is required for peptidoglycan hydrolysis during chloroplast division
Source: BMC Plant Biol. 2014 Mar 6;14:57. doi: 10.1186/1471-2229-14-57 (PMC4015805; doi:10.1186/1471-2229-14-57)
Supplement: Additional file 1: Table S1 — GenInfo Identifier (GI) numbers or locus IDs of the amino acid or nucleotide sequences of cyanobacterial and chloroplast division proteins. [file 1471-2229-14-57-S1.doc]

| Table S1. GenInfo Identifier (GI) numbers or locus IDs of the amino acid or nucleotide sequences of cyanobacterial and chloroplast division proteins. | | | | | | | | | | | | |
| --- | --- | --- | --- | --- | --- | --- | --- | --- | --- | --- | --- | --- |
|  | Proteins | | | | | | | | | | | |
| Species | FtsZ | Ftn2/ARC6 | SepF | FtsQ | Ftn6 | MinC | MinD | MinE | FtsI | FtsW | AmiA | DipM |
| *S. elongatus* | Synpcc7942  2378 | Synpcc7942  1943 | Synpcc7942  2059 | Synpcc7942  2377 | Synpcc7942  1707 | Synpcc7942  2001 | Synpcc7942  0896 | Synpcc7942  0897 | Synpcc7942  0482 | Synpcc7942  0324 | Synpcc7942  2360 | Synpcc7942  0598 |
| *C. paradoxa* | GI:66954464 | Contig37232 | GI:1351796 |  |  |  | GI:383212634 | GI:383212636 | Contig11029 Contig15041 | GI:1016213 (CP) |  | Contig38889 |
| *C. merolae* | CMO089C CMS004C |  |  |  |  |  |  |  |  |  |  |  |
| *C. reinhardtii* | GI: 158272260 GI: 159488863 | GI:158279603 |  |  |  | GI:159469133 | GI:159477869 | GI:159478206 |  |  |  |  |
| *P. patens* | GI: 162668292 GI: 168033107 GI: 162679692 GI: 162682859 GI: 162681984 GI: 168025380 |  |  |  |  | GI:168012958 | GI:168021283 GI:168010508 | GI:162687254 GI:168001327 GI:168059874 |  |  |  | GI:567757316 (DipM1) GI:168028139 (DipM2)  GI:168061355  GI:168053993 |
| *S. moellendorffii* | GI: 302765324 GI:302806196 GI:302808417 GI:302789456 GI:302783276 | GI:302773209 GI:302823327 |  |  |  |  | GI:302755989 | GI:302768475 GI:302761908 GI:302786540 GI:302791389 |  |  |  | GI:169112254 (EST) |
| *A. thaliana* | At2g36250 At3g52750 At5g55280 | At5g42480 At3g19180 |  |  |  |  | At5g24020 | At1g69390 |  |  |  |  |
