# Supplementary figures and images for: DipM is required for peptidoglycan hydrolysis during chloroplast division
Source: BMC Plant Biol. 2014 Mar 6;14:57. doi: 10.1186/1471-2229-14-57 (PMC4015805; doi:10.1186/1471-2229-14-57)

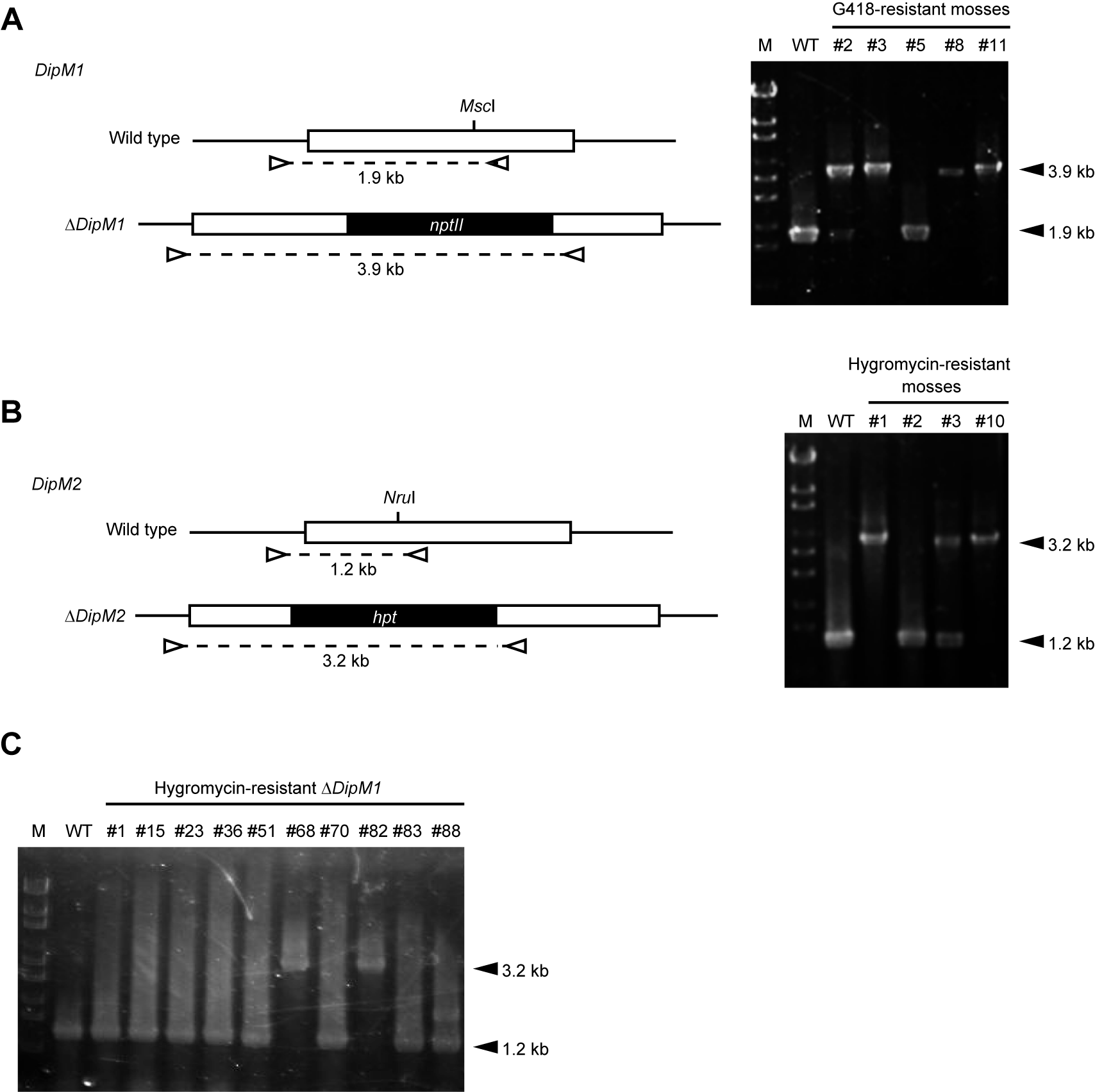

Supplement: Additional file 2: Figure S1 — Confirmation of DipM1 and DipM2 disruption in P. patens. (A) Insertional mutation of the P. patens DipM1 locus. The nptII gene was inserted into the DipM1 locus and the insertion was detected by PCR. The PCR resulted in 3.9-kbp or 1.9-kbp products from inserted or intact chromosomes, respectively. #3, #8, and #11 were used for further analyses. (B) Insertional mutation of P. patens DipM2 locus. hpt gene was inserted into DipM2 locus and the insertion was detected by PCR. The PCR produces 3.2-kbp or 1.2-kbp products from inserted or intact chromosomes, respectively. #1 and #10 were used for further analyses. (C) hpt gene was inserted into DipM2 locus of ∆DipM1 mutant. The insertion was checked by PCR as in (B). #68 and #82 were used for further analyses. [file 1471-2229-14-57-S2.png]
